# Long Non-coding RNA FGD5-AS1 Regulates Cancer Cell Proliferation and Chemoresistance in Gastric Cancer Through miR-153-3p/CITED2 Axis

**DOI:** 10.3389/fgene.2020.00715

**Published:** 2020-08-11

**Authors:** Yunhan Gao, Mubing Xie, Yi Guo, Qian Yang, Song Hu, Zhongfu Li

**Affiliations:** ^1^Department of General Surgery, Chongqing University Central Hospital, Chongqing Emergency Medical Center, The Fourth People’s Hospital of Chongqing, Chongqing, China; ^2^Department of General Surgery, Yongchuan Dakang Hospital of Traditional Chinese Medicine, Chongqing, China

**Keywords:** gastric cancer, lncRNA, FGD5-AS1, miRNA, miR-153-3p, CITED2

## Abstract

**Background:**

In this study, we investigated the molecular mechanisms of human long non-coding RNA (lncRNA) FYVE RhoGEF And PH Domain Containing 5 Antisense RNA 1 (FGD5-AS1) and its downstream epigenetic axis, human microRNA-153-3p (hsa-miR-153-3p)/Cbp/P300-interacting transactivator with Glu/Asp-rich carboxy-terminal domain 2 (CITED2) in human gastric cancer.

**Methods:**

Gastric cancer cell lines and clinical tumor samples were used to assess FGD5-AS1 expression levels. Lentivirus containing FGD5-AS1 small interfering RNA (sh-FGD5AS1) was applied to knockdown FGD5-AS1 expression. Cancer cells *in vitro* and *in vivo* proliferation, and 5-FU chemoresistance were assessed, respectively. Expressions of hsa-miR-153-3p/CITED2 were also assessed in FGD5-AS1-downregulated gastric cancer cells. Hsa-miR-153-3p was knocked down and CITED2 was upregulated to assess their direct functional correlations with FGD5-AS1 in gastric cancer.

**Results:**

Both gastric cancer cell lines and human tumor samples showed aberrant FGD5-AS1 upregulation. Lentiviral-induced FGD5-AS1 knockdown reduced cancer proliferation, 5-FU chemoresistance *in vitro*, and tumorigenicity *in vivo*. Hsa-miR-153-3p/CITED2 axis was confirmed to be downstream of FGD5-AS1 in gastric cancer. Hsa-miR-153-3p inhibition or CITED2 upregulation reversed the tumor-suppressing effects of FGD5-AS1 downregulation on gastric cancer proliferation and 5-FU chemoresistance.

**Conclusion:**

We demonstrated that FGD5-AS1 can regulate human gastric cancer cell functions, possibly through its downstream epigenetic axis of hsa-miR-153-3p/CITED2.

## Introduction

Gastric cancer is one of the most aggressive malignancies and is a leading cause of cancer-related death in East Asia (Leung et al., 2008; Yamaoka et al., 2008). In China, gastric cancer patients are often diagnosed at late stages with very low 5-year survival rates (∼ < 20%) (Yang, 2006; Chen et al., 2016). In addition, late-stage gastric tumors often metastasize to adjacent or even distant organs in the body, such as the liver, lungs, bones, or lymph nodes, thus burdening gastric cancer patients with poor prognosis and limited treatment options (Gotoda et al., 2000; Kim et al., 2014). It is therefore very important to seek new genetic pathways underlying cancer oncogenesis and development, in order to develop early diagnostic methods and efficient treatments for patients with gastric cancer.

Recently, long non-coding RNAs (lncRNAs), long-length (∼ > 200 nt), and non-coding RNAs, have emerged as important transcripts to regulate biological functions in human cells (Esteller, 2011; Harries, 2012). Specifically, lncRNAs were demonstrated to be aberrantly expressed in many types of human cancers and exhibited either oncogenic or tumor-suppressing effects on modulating cancer oncogenesis, maturation, metastasis, and apoptosis (Du et al., 2013; Yan et al., 2015). Moreover, several groups of lncRNAs had been identified to be closely associated with human gastric cancer (Li et al., 2014, 2016). Among them, lncRNA of FYVE RhoGEF And PH Domain Containing 5 Antisense RNA 1 (FGD5-AS1) had been shown to be overexpressed, possibly acting as an oncogenic promotor in oral cancer, colorectal cancer, and lung cancer (Li et al., 2019; Fan et al., 2020; Liu et al., 2020). However, the expression profile and molecular function of FGD5-AS1 in human gastric cancer have never been elucidated.

MicroRNAs (miRNAs) are another group of non-coding RNAs but with short-length (18–22 nt). It has been shown that miRNAs may physically bind the complementary sites in the 3′-untranslated region (3′-UTR) of downstream target transcripts to induce post-transcriptional gene suppression or protein degradation (Carrington and Ambros, 2003). In addition, emerging evidence has suggested that miRNAs may act as the competing endogenous RNA (ceRNA) of lncRNAs, thus further extending the epigenetic regulation among human diseases (Shi et al., 2013; Sanchez-Mejias and Tay, 2015). In human gastric cancer, it has been demonstrated that miRNAs may either directly act through its downstream signaling pathway, or is indirectly associated with lncRNAs to modulate cancer cell development (Shrestha et al., 2014; Xia et al., 2014). Among them, human microRNA 153 (hsa-miR-153) has been demonstrated to be aberrantly expressed in gastric cancer tumor tissues and function as tumor suppressors or biomarkers (Wang and Liu, 2015; Zhang et al., 2015; Ouyang et al., 2018). However, it is largely unknown whether any upstream lncRNAs may be associated with miR-miR-153 to regulate gastric cancer cell functions.

In the current study, we first examined the gene expression of FGD5-AS1 in gastric cancer cell lines and clinical samples. We hypothesized that the downregulation of FGD5-AS1 would suppress gastric cancer cell proliferation and chemoresistance. We also hypothesized that hsa-miR-153-3p, as well as its downstream gene candidate, Cbp/P300-interacting transactivator with Glu/Asp-rich carboxy-terminal domain 2 (CITED2), may serve as the ceRNA candidate to be correlated with FGD5-AS1 in regulating gastric cancer cell proliferation and chemoresistance.

## Materials and Methods

### Ethics Statement

In our study, all experimental protocols were approved by the Human Research Boards and Ethics Committees at the Chongqing University Central Hospital and the Yongchuan Dakang Hospital of Traditional Chinese Medicine Chongqing Medical University in Chongqing, China. All experiments were carried out in accordance with the principles of the Declaration of Helsinki. All participating patients signed consent forms prior to the onset of this study.

### Gastric Cancer Cell Lines and Clinical Samples

Five gastric cancer cell lines, AGS, NIC-N87, SNU-1, SNU-5, and SNU-16 were purchased from the American Type Culture Collection (ATCC, United States). The other four gastric cell lines, MKN-45, SGC-7901, BGC-823, and MKN-28, and a normal gastric epithelial cell line GES-1, were purchased from the cell bank of the Type Culture Collection of the Chinese Academy of Sciences (Shanghai, China). All cell lines were maintained in DMEM medium (Thermo Fisher Scientific, United States), supplemented with 10% fetal bovine serum (FBS, MilliporeSigma, Shanghai, China), 100 μg/mL streptomycin (Thermo Fisher Scientific, United States), and 100 U/mL penicillin (Thermo Fisher Scientific, United States) in a tissue incubator of 5% CO_2_ at 37°C. Media changes were performed every 2–3 days (70–80% confluence).

Gastric cancer clinical samples, including paired gastric tumor tissues and adjacent non-tumor tissues (at least 3 cm away from the clear edge of tumor tissues) were surgically extracted from 45 patients in the departments of the General surgery at Chongqing University Central Hospital, the Fourth People’s Hospital of Chongqing, and the Yongchuan Dakang Hospital of Traditional Chinese Medicine in Chongqing, China. At the time of tissue collection, none of the patients received any adjuvant treatments, thus eliminating treatment-induced gene changes. Then, all clinical samples were immediately snap-frozen in liquid nitrogen and stored at −80°C until further processing.

### RNA Extraction and Quantitative RT-PCR

RNA extraction and quantitative RT-PCR (qRT-PCR) was carried out according to the methods in our previous publications with slight modifications (Jian et al., 2016; Tang et al., 2017). Briefly, total RNA from gastric cancer cell lines and clinical samples was isolated with a RNeasy mini kit (Thermo Fisher Scientific, United States) according to the manufacturer’s instructions. After verifying the purity of RNA, cDNA was reversely transcribed trough a M-MLV-RTase kit (Promega, Sunnyvale, CA) and probed on an Applied Biosystems thermal cycler (ABI 2720; ABI Biosystems, United States) according to the manufacturer’s instructions. To detect the FGD5-AS1 expression level, a pre-designed Taqman human non-coding RNA assay (ABI Biosystems, United States) was used with 18s transcript as the loading template. To detect hsa-miR-153-3p, a pre-designed TaqMan human miRNA Assay (Applied Biosystem, United States) was used with U6 transcript as the loading template. To detect the CITED2 expression level, a SYBR Green Real-Time PCR Master Mix Kit (Applied Biosystems, United States) was used with GAPDH as the loading template. All final qRT-PCR results were reported using the 2^–ΔΔCt^ calculation.

### FGD5-AS1 Downregulation Assay

A lentivirus containing human lncRNA FGD5-AS1 short hairpin RNA (shRNA) (sh_FGD5AS1), and a lentivirus containing negative control shRNA (sh-NC) were purchased from SunBio (SunBio Medical Biotechnology, Shanghai, China). Gastric cancer cell lines, SGC-7901, and MKN-28 cells were seeded in 96-well plates (7,500 cells/well) and transduced with 100 pmol lentiviruses, along with 8 μg/mL polybrene (MilliporeSigma, Shanghai, China), at a multiplicity of infection (MOI) of 2–5 for 72 h, followed by a selection procedure of G418 (500 μg/mL, MilliporeSigma, Shanghai, China) for 48 h. Then, healthy cells were collected and maintained for another 5–7 days to stabilize the lentivirus transduction. After that, the transduction efficiency was verified by qRT-PCR.

### Cell Proliferation Assay

The cancer cell proliferation assay was carried out according to the methods in our previous publication (Jian et al., 2016). Briefly, SGC-7901 and MKN-28 cells were seeded in 96-well plates (5,000 cells/well). A 3-(4,5-dimethylthiazol-2-yl)-2,5-diphenyltetrazolium bromide (MTT) assay (Thermo Fisher Scientific, United States) was then applied every 24 h for 5 days. Cancer cell proliferating rates were measured as the optical density (OD) at a wavelength of 570 nm using an FL500 microplate fluorescence reader (Bio-Tek, United States) according to the manufacturer’s instructions.

### Chemoresistance Assay

SGC-7901 and MKN-28 cells were seeded in 96-well plates (7,500 cells/well). Chemo-drug of Fluorouracil (5-FU) (MilliporeSigma, Shanghai, China) was added into the cell culture, at concentrations of 0, 5, 10, 20, 50, and 100 μg/mL for 24 h. Cancer cell viability was measured using an MTT assay and normalized to the value for cells treated with 0 μg/mL 5-FU.

### *In vivo* Tumorigenicity Assay

The *in vivo* tumorigenicity assay was carried out according to the methods in our previous publication with slight modification (Jian et al., 2016). Briefly, lentiviral-transduced SGC-7901 cells were subcutaneously injected into the flanks of 2-month old athymic mice (a total of eight mice). The left flanks were injected with cells transduced with sh-FGD5AS1 and the right flanks with cells transduced with sh-NC. For 5 consecutive weeks, *in vivo* tumor volumes (mm^3^) were measured using the formula, length × width^2^/2. At the end of the tumorigenicity assay, mice were sacrificed and SGC-7901 tumors were extracted and compared.

### Dual-Luciferase Reporter Assay

The dual-luciferase reporter assay was carried out according to the methods in our previous publication with slight modification (Jian et al., 2016). Briefly, wild type (WT) 3′-UTRs of human FGD5-AS1 and CITED2, both enclosing the putative hsa-miR-153-3p binding sites, were sub-cloned into psiCHECK2 luciferase plasmid (Promega, United States) and named as FGD5AS1-WT and CITED2-WT, respectively. Alternatively, hsa-miR-153-3p binding sites were mutated on both FGD5-AS1 and CITED2 3′-UTRs and the mutant (MT) 3′-UTRs were sub-cloned into psiCHECK2, and named as FGD5AS1-MT and CITED2-MT, respectively. In the culture of human HEK293T, cells were co-transfected with either FGD5AS1-WT, FGD5AS1-MT, CITED2-WT, or CITED2-MT, along with either hsa-miR-153-3p mimics (miR-153-3p-mimics, RiboBio, Guangzhou, China) or non-specific miRNA mimics (miR-NC, RiboBio, Guangzhou, China) for 48 h. A dual-luciferase reporter assay (Promega, United States) was then carried out according to the manufacturer’s instructions.

### Hsa-miR-153-3p Downregulation Assay

A synthesized hsa-miR-153-3p inhibitor (miR-153-In) and a negative control human miRNA inhibitor (miR-NC) were purchased from SunBio (SunBio Medical Biotechnology, Shanghai, China). SGC-7901 and MKN-28 cells were transfected with miR-153-In or miR-NC for 48 h, followed by a qRT-PCR assay to confirm the downregulation efficacy.

### CITED2 Overexpression Assay

A mammalian expression plasmid (pcDNA 3.1/+) containing the whole cDNA sequence of human CITED2 (pc/CITED2) and an empty control pcDNA 3.1/+ plasmid (pc/C) were purchased from RiboBio (RiboBio, Guangzhou, China). SGC-7901 and MKN-28 cells were transfected with pc/CITED2 or pc/C for 48 h, followed by a qRT-PCR assay to confirm the overexpression efficacy.

### Statistical Analysis

All experiments were biologically repeated at least three times. All data are presented as mean and standard errors. Differences were determined using one-way ANOVA with a *post hoc* test (SPSS v19; IBM, Armonk, NY). Differences were considered statistically significant if *P* < 0.05.

## Results

### FGD5-AS1 Gene Levels Are Upregulated in Gastric Cancer Lines and Tumor Samples

**FIGURE 1:**
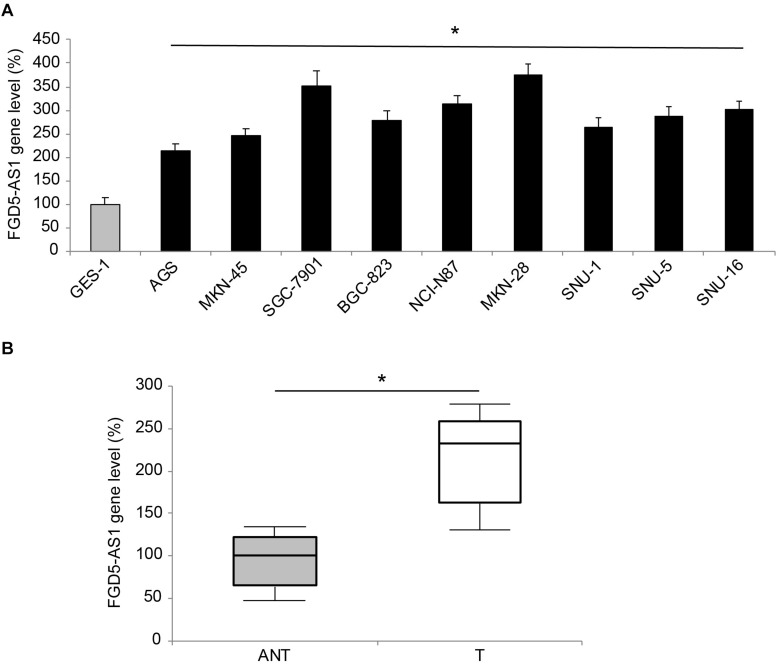
Expression of FGD5-AS1 in gastric cancer. **(A)** FGD5-AS1 gene levels were measured, by qRT-PCR, in gastric cancer cell lines of AGS, MKN-45, SGC-7901, BGC-823, NCI-N87, MKN-28, SNU-1, SNU-5, and SNU-16, and then compared to the FGD5-AS1 gene level in a normal gastric epithelial cell line, GES-1 (*P < 0.05, vs. GES-1). **(B)** In 45 patients diagnosed with gastric cancer, FGD5-AS1 expression was compared between gastric tumor (T) samples and adjacent non-tumor (ANT) gastric tissue samples (*P < 0.05).

Gene expression levels of human lncRNA FGD5-AS1 were measured in nine gastric cancer cell lines, and a normal gastric epithelial cell line, GES-1. The results of qRT-PCR showed significant upregulations of FGD5-AS1 in all tested gastric cancer cells, as compared to GES-1 (Figure 1A, *P < 0.05). FGD5-AS1 gene levels were also compared between gastric tumor samples (T) and their adjacent non-tumor (ANT) gastric tissues in 45 patients diagnosed with gastric cancer. Again, qRT-PCR demonstrated that FGD5-AS1 was markedly upregulated in tumor samples compared to non-tumor gastric tissues (Figure 1B, *P < 0.05).

### Downregulation of FGD5-AS1 Suppressed Gastric Cancer Cell Proliferation and Chemoresistance

**FIGURE 2:**
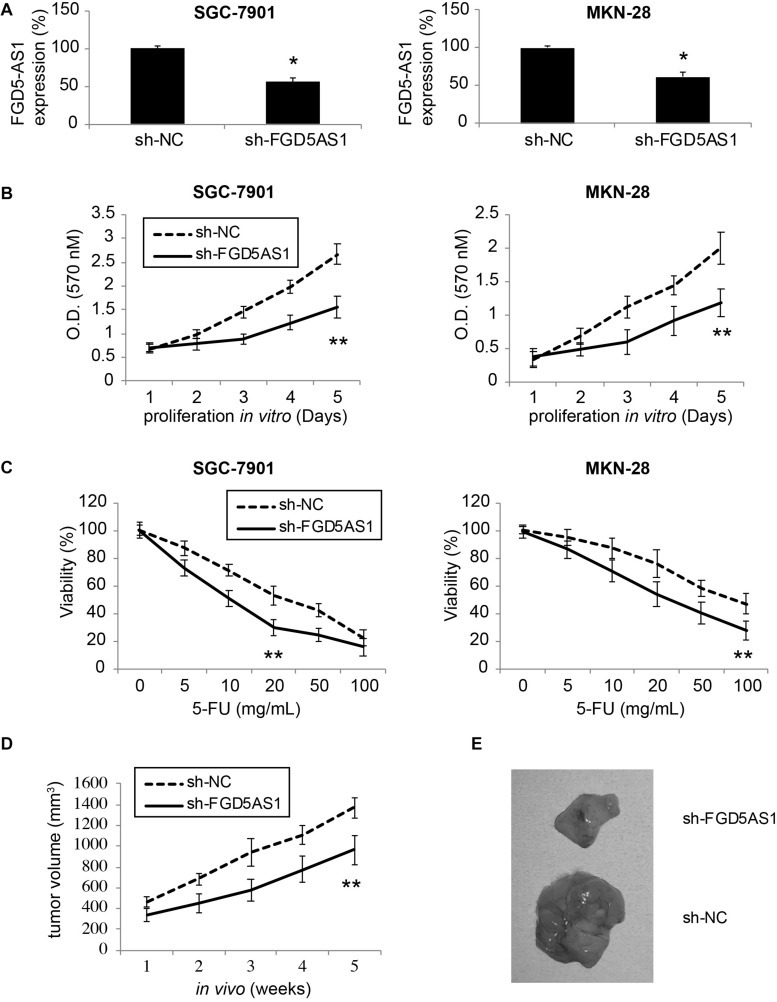
Effects of FGD5-AS1 inhibition on gastric cancer cell proliferation and 5-FU chemoresistance. **(A)** Two gastric cancer cell lines, SGC-7901 and MKN-28 were transduced with a lentivirus containing human lncRNA FGD5-AS1 sh_RNA (sh_FGD5AS1), or a lentivirus containing negative control shRNA (sh-NC). After transduction was stabilized, qRT-PCR was applied to compare FGD5-AS1 expression levels (*P < 0.05). **(B)** A 5-day MTT assay was used to assess *in vitro* proliferation rates between SGC-7901 and MKN-28 cells transduced with sh-NC and those transduced with sh-FGD5AS1 (**P < 0.05). **(C)** Lentiviral-transduced SGC-7901 and MKN-28 cells were treated with 0, 5, 10, 20, 50, or 100 μg/mL 5-FU for 24 h, followed by an MTT assay to compare 5-FU chemoresistance between those transduced with sh-NC and those transduced with sh-FGD5AS1 (**P < 0.05). **(D)** Lentiviral-transduced SGC-7901 cells were subcutaneously inoculated (1 × 10^6^/injection) into the flanks of 2-month-old female athymic mice for 5 weeks. The *in vivo* tumor volumes (mm^3^) were compared between those transduced with sh-NC and those transduced with sh-FGD5AS1 (**P < 0.05). **(E)** After the *in vivo* tumorigenicity assay, SGC-7901 tumors were retrieved and compared directly.

Two gastric cancer cell lines, SGC-7901 and MKN-28, were transduced with lentiviruses of sh-NC or sh-FGD5AS1. After transduction was stabilized, qRT-PCR confirmed that in both SGC-7901 and MKN-28 cells, FGD5-AS1 expressions were significantly downregulated by transduction of sh-FGD5AS1, as compared to sh-NC (Figure 2A, *P < 0.05).

A 5-day MTT assay was then carried out to compare cancer proliferation among lentiviral- transduced SGC-7901 and MKN-28 cells. It demonstrated that in cells transduced with sh-FGD5AS1, cancer cell proliferation rates were significantly suppressed, as compared to cells transduced with sh-NC (Figure 2B, **P < 0.05).

In addition, lentiviral-transduced SGC-7901 and MKN-28 cells were treated with series concentrations of 5-FU (0, 5, 10, 20, 50, and 100 μg/mL) for 24 h. It showed that 5-FU chemoresistance was significantly reduced in cells transduced with sh-FGD5AS1, as compared to cells transduced with sh-NC (Figure 2C, **P < 0.05).

Moreover, we investigated the effect of FGD5-AS1 downregulation on gastric cancer *in vivo* tumor growth. SGC-7901 cells transduced with sh-NC or sh-FGD5AS1 were subcutaneously injected into the flanks of 2-month old athymic mice. Cells were grown for 5 weeks, and the *in situ* tumor volumes were estimated weekly. It demonstrated that gastric cancer cell *in vivo* proliferation was severely hindered in SGC-7901 tumors transduced with sh-FGD5AS1, as compared to tumors transduced with sh-NC (Figure 2D, **P < 0.05). At the end of the *in vivo* tumorigenicity assay, visual examination on extracted SGC-7901 tumors confirmed this observation, demonstrating distinct size differences between sh-FGD5AS- and sh-NC-transduced tumors (Figure 2E).

These results therefore clearly indicated that downregulation of FGD5-AS1 had significantly anti-tumor effects on gastric cancer proliferation (both *in vitro* and *in vivo*) and chemoresistance response to 5-FU.

### Hsa-miR-153-3p/CITED2 Axis Is Correlated With FGD5-AS1 in Gastric Cancer

**FIGURE 3:**
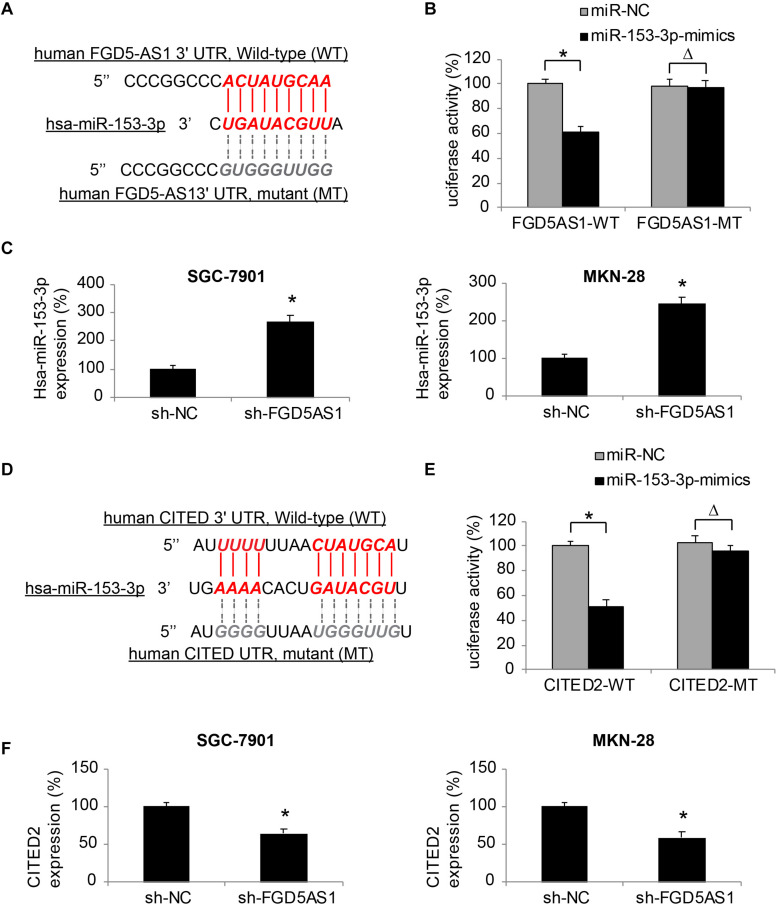
Effects of FGD5-AS1 on hsa-miR-153-3p/CITED2 expression in gastric cancer. **(A)** A DNA skeleton map is demonstrated to show the putative binding of human FGD5-AS1 3′-UTR on hsa-miR-153-3p (Red). A mutated FGD5-AS1 3′-UTR sequence is also shown to nullify the binding of hsa-miR-153-3p (Gray). **(B)** HEK293T cells were co-transfected with luciferase plasmids with wild type human FGD5-AS1 3′UTR (FGD5AS1-WT) or with mutant FGD5-AS1 3′UTR (FGD5AS1-MT), and miR-153-3p-mimics or miR-NC for 48 h. The relative luciferase activities were measured by a dual-luciferase assay (*P < 0.05, Δ*P* > 0.05). **(C)** In lentiviral-transduced SGC-7901 and MKN-28 cells, hsa-miR-153-3p expression levels were compared between the cells transduced with sh-FGD5AS1 and those transuded with sh-NC (*P < 0.05). **(D)** Another DNA skeleton map demonstrates the putative binding of human CITED2 3′-UTR on hsa-miR-153-3p (Red). A mutated CITED2 3′-UTR sequence is shown to nullify the binding of hsa-miR-153-3p (Gray). **(E)** HEK293T cells were co-transfected with luciferase plasmids with wild type human CITED2 3′UTR (CITED2-WT) or with mutant CITED2 3′UTR (CITED2-MT), and miR-153-3p-mimics or miR-NC for 48 h. The relative luciferase activities were measured by a dual-luciferase assay (*P < 0.05, Δ*P* > 0.05). **(F)** In lentiviral-transduced SGC-7901 and MKN-28 cells, CITED2 expression levels were compared between the cells transduced with sh-FGD5AS1 and those transuded with sh-NC (*P < 0.05).

Through bioinformatic research using online epigenetic databases, such as TargetScan (Agarwal et al., 2015) and DIANA-LncBase (Paraskevopoulou et al., 2016), we discovered that human mature microRNA-153-3p (hsa-miR-153-3p) may serve as a possible downstream competing RNA of FGD5-AS1 (Figure 3A).

We thus created two luciferase plasmids; one (FGD5AS1-WT) included the WT 3′-UTR of human FGD5-AS1, which hosted a putative hsa-miR-153-3p binding site, the other (FGD5AS1-MT) included a mutated (MT) 3′-UTR of human FGD5-AS1 to nullify the miR-153-3p binding site. FGD5AS1-WT or FGD5AS1-MT was then co-transfected with either miR-153-3p-mimics or miR-NC in human HEK293T cells. Forty-eight hours later, the result of dual-luciferase activity assay showed that, there was a significant luciferase activity decrease in KEK293T cells co-transfected with FGD5AS1-WT and miR-153-3p-mimics (Figure 3B, *P < 0.05), thus confirming that hsa-miR-153-3p was the competing RNA of FGD5-AS1.

In addition, the effect of FDG5-AS1 downregulation on hsa-miR-153-3p expression in gastric cancer cells was investigated by qRT-PCR. It indicated that in lentiviral-transduced SGC-7901 and MKN-28 cells, hsa-miR-153-3p expressions were significantly upregulated by FGD5-AS1 downregulation (Figure 3C, *P < 0.05).

Moreover, we investigated the possible downstream transcript of hsa-miR-153-3p. Also using the bioinformatic approach, we identified that the human CITED2 gene was very likely a downstream candidate of hsa-miR-153-3p (Figure 3D).

We constructed another set of luciferase plasmids; one (CITED2-WT) included the WT 3′-UTR of human CITED2 gene, which hosted a putative hsa-miR-153-3p binding site, the other (CITED2-MT) included a mutated (MT) 3′-UTR of the human CITED2 gene to nullify the miR-153-3p binding site. A dual-luciferase activity assay then demonstrated that CITED2 could be bound by hsa-miR-153-3p (Figure 3E, *P < 0.05).

Finally, qRT-PCR experiments showed that in lentiviral-transduced SGC-7901 and MKN-28 cells, CITED2 expressions were markedly suppressed by FGD5-AS1 downregulation (Figure 3F, *P < 0.05).

These results therefore strongly suggested that FGD5-AS1 regulated an epigenetic downstream axis of hsa-miR-153-3p/CITED2 in human gastric cancer.

### Downregulation of hsa-miR-153-3p Reversed the Tumor-Suppressive Functions of FGD5-AS1 Downregulation in Gastric Cancer

**FIGURE 4:**
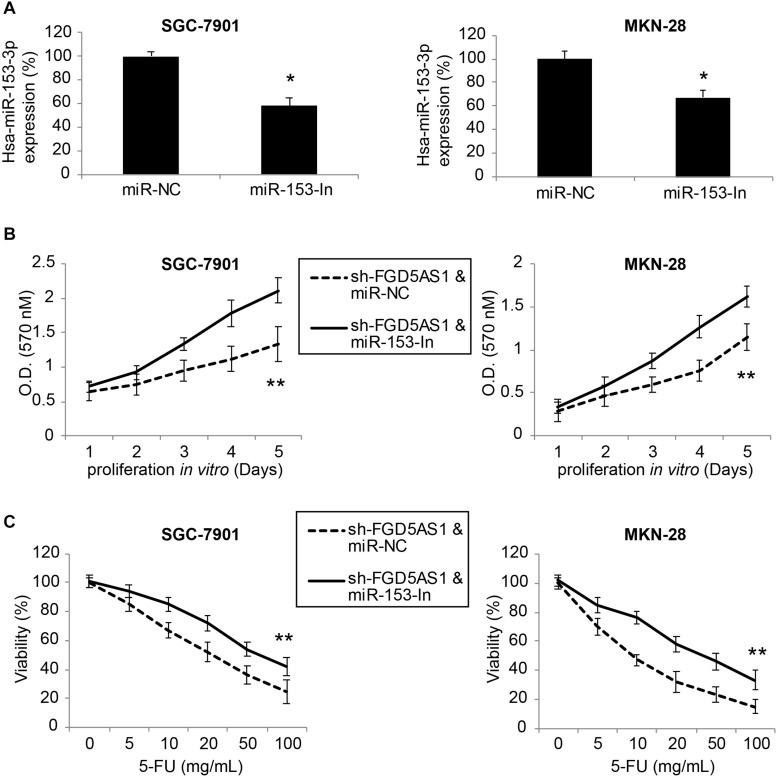
Inhibiting hsa-miR-153-3p restored cancer cell proliferation and chemoresistance in gastric cancer cells with FGD5-AS1 downregulation. **(A)** In sh-FGD5AS1-transduced SGC-7901 and MKN-28 cells, a synthesized hsa-miR-153-3p inhibitor (miR-153-In), or a negative control miRNA inhibitor (miR-NC) was transfected into cells. After 48 h, qRT-PCR was carried out to verify the transfection efficiency (*P < 0.05). **(B)** A 5-day MTT assay was carried out to compare the proliferation rates between SGC-7901 and MKN-28 cells infected with sh-FFG5AS1 & miR-NC, and those infected with sh-FFG5AS1 and miR-153-In (**P < 0.05). **(C)** In double-infected SGC-7901 and MKN-28 cells, 5-FU chemoresistance was compared between cells infected with sh-FFG5AS1 & miR-NC, and those infected with sh-FFG5AS1 and miR-153-In (**P < 0.05).

For SGC-7901 and MKN-28 cells transduced with sh-FGD5AS1, we further transfected them with either miR-153-In or miR-NC for 48 h. After that, qRT-PCR confirmed that hsa-miR-153-3p expression was significantly downregulated by miR-153-In transfection in both gastric cancer cell lines (Figure 4A, *P < 0.05).

We then re-assessed cancer proliferation and chemoresistance in double-infected SGC-7901 and MKN-28 cells. Using the MTT assay, we discovered that gastric cancer cell proliferating rates were significantly accelerated in cells double-infected with sh-FGD5AS1 and miR-153-In, compared to cells double-infected with sh-FGD5AS1 and miR-NC (Figure 4B, **P < 0.05). Using the 5-FU chemoresistance assay, we also discovered that drug resistance was markedly increased in cells double-infected with sh-FGD5AS1 and miR-153-In, compared cells double-infected with sh-FGD5AS1 and miR-NC (Figure 4C, **P < 0.05).

### Upregulation of CITED2 Also Reversed the Tumor-Suppressive Functions of FGD5-AS1 Downregulation in Gastric Cancer

**FIGURE 5:**
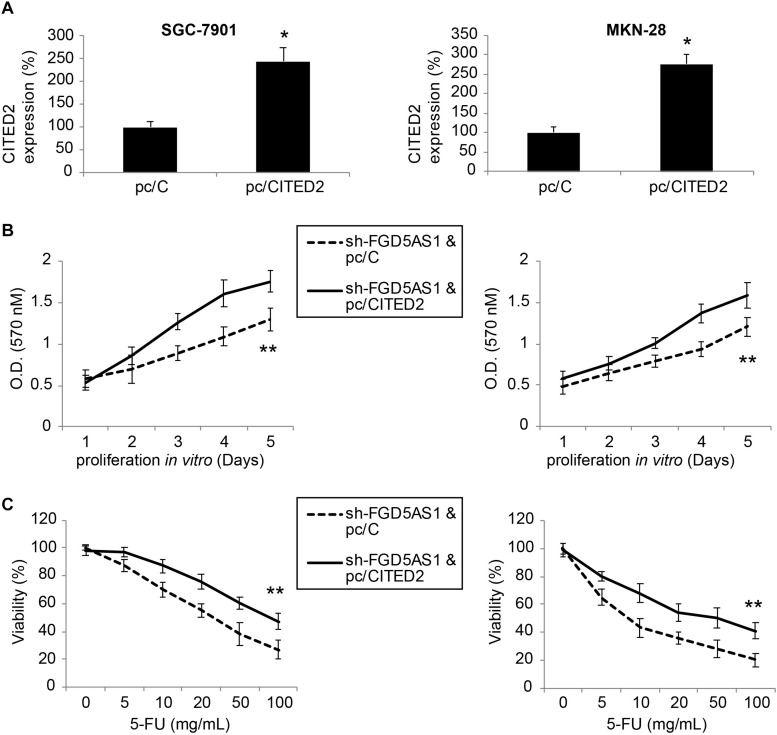
Overexpressing CITED2 also restored cancer cell proliferation and chemoresistance in gastric cancer cells with FGD5-AS1 downregulation. **(A)** In sh-FGD5AS1-transduced SGC-7901 and MKN-28 cells, a mammalian expression plasmid containing the whole cDNA sequence of human CITED2 (pc/CITED2), or an empty control pcDNA 3.1/+ plasmid (pc/C), was transfected into cells. After 48 h, qRT-PCR was carried out to verify the transfection efficiency (*P < 0.05). **(B)** A 5-day MTT assay was carried out to compare the proliferation rates between SGC-7901 and MKN-28 cells infected with sh-FFG5AS1 and pc/C, and those infected with sh-FFG5AS1 and pc/CITED2 (**P < 0.05). **(C)** In double-infected SGC-7901 and MKN-28 cells, 5-FU chemoresistance was compared between cells infected with sh-FFG5AS1 and pc/C, and those infected with sh-FFG5AS1 and pc/CITED2 (**P < 0.05).

Finally, sh-FGD5AS1-transduced SGC-7901 and MKN-28 cells were transfected with either pc/CITED2 or pc/C for 48 h. After that, qRT-PCR indicated that CITED2 expression was markedly upregulated by pc/CITED2 transfection in gastric cancer cell lines (Figure 5A, *P < 0.05).

We again re-assessed cancer proliferation and chemoresistance in these double-infected SGC-7901 and MKN-28 cells. Using the MTT assay, we discovered that gastric cancer cell proliferating rates were significantly accelerated in cells double-infected with sh-FGD5AS1 and pc/CITED2, compared to cells double-infected with sh-FGD5AS1 and pc/C (Figure 5B, **P < 0.05). With the 5-FU chemoresistance assay, it was shown that drug resistance was markedly increased in cells double-infected with sh-FGD5AS1 and pc/CITED2, compared to cells double-infected with sh-FGD5AS1 and pc/C (Figure 5C, **P < 0.05).

## Discussion

Emerging evidence has demonstrated that lncRNAs, along with their ceRNA candidates, may play critical roles in human gastric cancer (Li et al., 2014; Xia et al., 2014). In this study, we first demonstrated that gastric cancer cell lines expressed higher levels of lncRNA FGD5-AS1. Similarly, the upregulated expression pattern of FGD5-AS1 was found in primary gastric tumor samples. These findings are very encouraging, as it indicates that FGD5-AS1 may potentially serve as a prognostic biomarker for patients with gastric cancer, despite the heterogeneous nature of gastric tumorigenesis, development, and metastasis (Nadauld and Ford, 2013; Hudler, 2015). Thus, further studies focusing on the correlation between FGD5-AS1 expression and cancer patients’ clinicopathological characteristics or clinical outcomes, may help to define its prognostic role in gastric cancer diagnosis.

Next in this study, we adapted genetic engineering to successfully generate two gastric cancer cell lines, SGC-7901 and MKN-28 with stable FGD5-AS1 downregulation. Using these cells, we were able to demonstrate that lentiviral-induced FGD5-AS1 downregulation markedly suppressed cancer cell proliferation, 5-FU chemoresistance, and *in vivo* tumorigenicity. Thus, our data indicated that FGD5-AS1 might be an oncogenic transcript in gastric cancer. This finding is certainly not surprising, as FGD5-AS1 was demonstrated to be overexpressed and to promote cancer cell maturation and metastasis in other human cancers, including colorectal cancer, lung cancer, and oral cancer (Li et al., 2019; Fan et al., 2020; Liu et al., 2020). However, our study is the first to report an aberrant expression and functional role of FGD5-AS1 in gastric cancer, further expending the understanding of overall epigenetic regulation of FGD5-AS1 in human cancer research.

Additionally, in this study, we identified an epigenetic axis of hsa-miR-153-3p/CITED2 to be the downstream ceRNA candidate of FGD5-AS1 in gastric cancer. Through a dual-luciferase assay, it was demonstrated that hsa-miR-153-3p could be found by FGD5-AS1 and CITED2. In addition, qRT-PCR showed that hsa-miR-153-3p expression was upregulated, whereas CITED2 was downregulated by FGD5-AS1 downregulation in gastric cancer cells. Most importantly, through further genetic engineering approaches, we demonstrated that miR-153-3p downregulation or CITED2 upregulation could directly reverse the inhibitory effect of FGD5-AS1 downregulation on gastric cancer proliferation and 5-FU chemoresistance. These results are consistent with those in past publications, as studies showed that miR-153 acts as a tumor suppressor to regulate gastric cancer proliferation and metastases (Wang and Liu, 2015; Ouyang et al., 2018), and our previous study showed that CITED2 was overexpressed in gastric cancer cells and its knockdown suppressed cell division, and promoted apoptosis in cancer cells (Tang et al., 2017). However, it is worth noting that in other types of human cancers, FGD5-AS1 was shown to exert its oncogenic modulation through other ceRNAs, such as miR-302e/CDCA7 axis in colorectal cancer (Li et al., 2019) or hsa-miR-107/FGFRL1 in lung cancer (Fan et al., 2020). These findings suggest that, in different types of human cancers, various downstream signaling pathways are likely to be involved in FGD5-AS1-associated epigenetic regulations. In addition, other miR-153 downstream transcripts, such as SNAI1 or KLF5 were reported to be involved in functional modulations in gastric cancer (Wang and Liu, 2015; Zhang et al., 2015; Ouyang et al., 2018). Thus, more investigations are needed to fully understand the epigenetic networks and their associated downstream pathways in functional regulations in gastric cancer.

Overall, we discovered new knowledge of FGD5-AS1’s aberrant overexpression and tumor-suppressing effects of its downregulation in human gastric cancer. In addition, we revealed that the epigenetic axis of hsa-miR-153-3p/CITED2 is directly correlated with FGD5-AS1 in regulating gastric cancer cell proliferation and 5-FU chemoresistance. These data may help in identifying new epigenetic pathways as therapeutic targets for patients with gastric cancer.

## Data Availability Statement

The raw data supporting the conclusions of this article will be made available by the authors, without undue reservation.

## Ethics Statement

The studies involving human participants were reviewed and approved by Human Research Boards and Ethics Committees at the Chongqing University Central Hospital and the Yongchuan Dakang Hospital of Traditional Chinese Medicine Chongqing Medical University in Chongqing, China. The patients/participants provided their written informed consent to participate in this study.

## Author Contributions

ZL: funding collection and final approval. ZL, YGa, and MX: manuscript construction. YGa and MX: manuscript written. YGa, QY, and SH: data analysis. All authors contributed to the article and approved the submitted version.

## Conflict of Interest

The authors declare that the research was conducted in the absence of any commercial or financial relationships that could be construed as a potential conflict of interest.
